# Differential modulation of rNa_V_1.4 channel inactivated states by lidocaine and its charged analogue QX222

**DOI:** 10.1186/1471-2210-11-S2-A30

**Published:** 2011-09-05

**Authors:** Péter Lukács, René Cervenka, Xaver Koenig, Ágnes Mike, Michael Kovar, Karlheinz Hilber, Hannes Todt

**Affiliations:** 1 Department of Neurophysiology and Neuropharmacology, Center for Physiology and Pharmacology, Medical University of Vienna, 1090 Vienna, Austria

## Background

The local anesthetic lidocaine is generally believed to reach its binding site in the intracellular vestibule of the voltage-gated sodium channel via the cell membrane. QX222 is a permanently charged, quaternary amine analogue of lidocaine, which can access this binding site via a hydrophilic route across the channel protein. The mutation I1575E of the adult rat muscle-type sodium channel (rNa_V_1.4) opens such a hydrophilic pathway. When bound to the internal vestibule, 500 µM lidocaine stabilize both fast and slow inactivated states. We have tested if QX222, once bound to the internal vestibule of I1575E mutant channel, exerts a similar modulatory action on inactivated states as lidocaine.

## Methods and results

The construct I1575E was transiently expressed in tsA201 cells and studied by means of the whole-cell patch-clamp technique. When applied from the extracellular side, 500 μM QX222 stabilized the slow but not the fast inactivated state in I1575E. When applied internally, QX222 entered the channel, but stabilization of inactivated states could not be observed. Position F1579 is the most important residue in local anaesthetic binding. Therefore we have tested I1575E/F1579A double mutant channels to see if QX222 modulates inactivation via this site. However, this mutant was insensitive to QX222.

## Conclusions

For both lidocaine and QX222 the binding site is in the inner vestibule of the channel, at position F1579. The hydrophilic form of lidocaine is responsible for block and stabilization of slow inactivation, whereas fast inactivation can only be stabilized by the hydrophobic form of lidocaine.

